# The Influence of Place on Weight Gain during Early Childhood: A Population-Based, Longitudinal Study

**DOI:** 10.1007/s11524-012-9712-8

**Published:** 2012-07-18

**Authors:** Megan Ann Carter, Lise Dubois, Mark S. Tremblay, Monica Taljaard

**Affiliations:** 1 Institute of Population Health, University of Ottawa, Ottawa, ON Canada; 2 Healthy Active Living and Obesity Research (HALO) CHEO Research Institute, Ottawa, ON Canada; 3 Ottawa Hospital Research Institute, Ottawa Hospital, Civic Campus, Ottawa, ON Canada

**Keywords:** Children, Neighborhood, Residential characteristics, Environment, Body weight, Body mass index, Longitudinal study, Mixed-models, Social factors

## Abstract

The objective of this paper was to determine the influence of place factors on weight gain in a contemporary cohort of children while also adjusting for early life and individual/family social factors. Participants from the Québec Longitudinal Study of Child Development comprised the sample for analysis (*n* = 1,580). A mixed-effects regression analysis was conducted to determine the longitudinal relationship between these place factors and standardized BMI, from age 4 to 10 years. The average relationship with time was found to be quadratic (rate of weight gain increased over time). Neighborhood material deprivation was found to be positively related to weight gain. Social deprivation, social disorder, and living in a medium density area were inversely related, while no association was found for social cohesion. Early life factors and genetic proxies appeared to be important in explaining weight gain in this sample. This study suggests that residential environments may play a role in childhood weight change; however, pathways are likely to be complex and interacting and perhaps not as important as early life factors and genetic proxies. Further work is required to clarify these relationships.

## Introduction

Childhood overweight and obesity have risen dramatically in the last 25 years in Canada^1^,^2^ and in many other countries.^3^,^4^ In 2004, 26 % of Canadian children aged 2–17 years were overweight and 8 % were obese.^2^ From 1978/1979 to 2004, the prevalence of overweight and obesity increased twofold among 6–17-year-olds. Due to this striking increase, as well as its potential for adversely affecting health both in the short and long term, being at excess weight during childhood has become a major global public health concern.

The etiologic literature on childhood weight status has tended to focus on individual characteristics rather than on broader contextual circumstances. The high prevalence of childhood obesity has not abated, suggesting that prevention strategies, traditionally implemented at the individual level, may not be effective. Conceptualizing childhood obesity within multiple levels of influence, specifically within residential communities and over the life course, is necessary to design effective prevention strategies that shift the distribution of risk downward. This is consistent with a social–ecological theory of health.^5^

Neighborhoods are a natural way to conceptualize “place” in terms of child health and acquiring health resources. In the context of obesity, complex interactions between individuals, families, local communities, and institutions, as well as the broader social environment, lead neighborhoods to become geographical areas that can: (1) structure opportunities/barriers for children to be physically active and to eat healthy^6^ and (2) give rise to negative perceptions, which themselves may elicit chronic stress responses leading to weight gain.^7^

Studies are starting to find significant relationships between different neighborhood characteristics and weight status;^8^ however, this is a fairly new area of research where the literature is heterogeneous and mostly cross-sectional.^9^ To better make the case for causation, longitudinal studies are needed that use measured heights and weights. Accounting for early life factors known to be related to childhood obesity development, as well as individual and family-level social factors and measures of the family environment can provide a more holistic picture of why and how weight status changes over time in young children.

Among the few longitudinal studies investigating the influence of place on childhood weight status, findings include significant negative relationships between change in BMI and area greenness/degree of vegetation,^10^ neighborhood income/deprivation,^11^ and perceived safety.^12^

Using the Québec Longitudinal Study of Child Development (QLSCD), the main objective of this study was to assess the influence of place factors on change in cohort children’s standardized weight for height while also accounting for other potentially important early life and individual-/family-level explanatory factors. The overall hypothesis was that unfavorable neighborhood characteristics such as high material and social deprivation, high social disorder, and low population density would be positively related to weight gain, while favorable characteristics such as high social cohesion would be inversely related to weight gain in children.

## Methods

### Sample

The QLSCD is a government-based cohort study conducted by the Institut de la Statistique du Québec (ISQ) to identify factors in early childhood that affect the health, social adjustment, and academic performance of young Quebeckers.^13^ The cohort is comprised of a representative sample of 2,120 children born in Québec in 1997/1998, who have been followed from 5 months of age.

**FIGURE 1.**
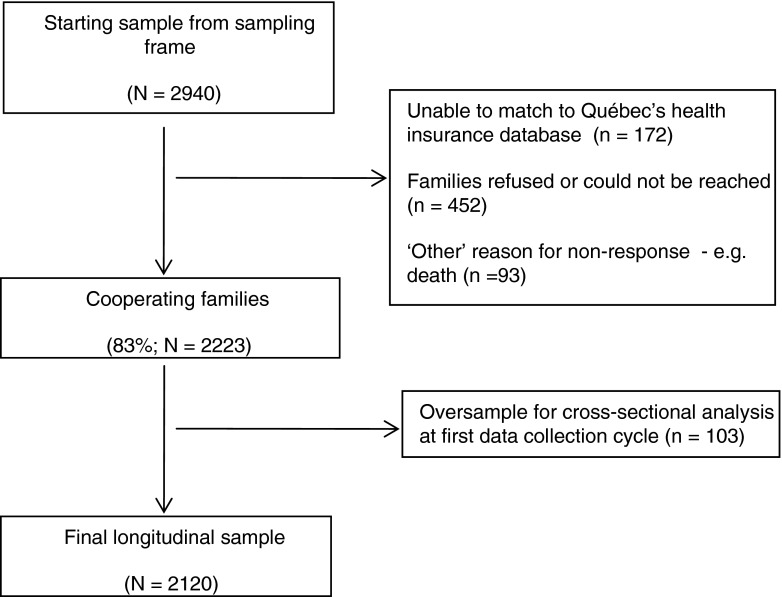
Sampling of children in the Québec Longitudinal Study of Child Development.

Cohort children were randomly selected based on a three-stage, stratified design.^14^ The territory covered by the survey was first divided into primary sampling units (PSUs) based on broad regions of Québec. The PSUs were then divided into two groups: remote or nonremote. In stage 1, two out of the four remote PSUs were chosen, and all 11 nonremote PSUs were chosen. The second stage involved dividing the selected regions into secondary sampling units (SSUs) based on one or two county regional municipalities. These were further divided into two groups: areas registering a high number of births in 1996 and those registering a low number of births. A fixed number of SSUs were randomly selected from the low birthrate group, and all SSUs were selected from the high birthrate group. Finally, in the third stage, a fixed number of children were randomly selected from the selected SSUs, based on the 1997/1998 Québec Birth Registry.^14^ Sampling occurred throughout the year to minimize the potential impact of seasonal influences (see Figure 1 for the sampling process). Twins, children with major diseases at birth, and those living in Northern Québec, Cree or Inuit territory, or Indian reserves were excluded.

From 5 months to 8 years of age, data collection occurred annually (timing changed slightly when children began school). In order to minimize respondent burden, this changed to a biannual basis from 8 years onward. Data from 5 months to 10 years are used in the present analysis. Computer-assisted personal interviewing of the mother, in the child’s home, was the primary method of data collection.^13^

## Variables

### Outcome

The primary outcome for this study was weight relative to height, as measured by BMI (kg/m^2^), standardized for age and sex using the Centers for Disease Control and Prevention Growth Charts, to obtain BMI Z-scores.^15^ The Z-score is the deviation of the value for an individual from the mean value of the reference population divided by the standard deviation for the reference population; in this case, the reference population has been derived from five different US surveys.^16^ The use of the BMI Z-score has been recommended as superior than percentiles for use in longitudinal analyses.^17^

Heights and weights were measured in the child’s home by trained interviewers at the approximate ages of 4, 6, 7, 8, and 10 years.^18^ At each data collection cycle, body weight was measured in kilograms on scales set back to zero for each measurement. Children wore light clothing and no shoes. Height was measured in meters.

### Main Exposures—Place Factors

#### Deprivation

Two forms of deprivation, material and social, were measured by an area-based index developed by Pampalon and Raymond,^19^ based largely on the work of Peter Townsend. The index was derived by linking postal codes of participants at data collection cycle 1, when children were approximately 5 months of age, to census data (1996) describing enumeration areas. Principal components analysis was used to create the index using six socioeconomic indicators: proportion of persons who have no high-school diploma; ratio of employment to total population; average income; proportion of persons who are separated, divorced, or widowed; proportion of people living alone; and proportion of single-parent families. The first three indicators form the material dimension of the index. This refers to the general inability of area residents to obtain the goods and conveniences that are a part of everyday life. The latter three indicators form the social dimension of the index, which refers to the fragmentation and weakening of the household structure. The index has been previously used to assess disparities in Canadian mortality rates^20^ and by the Québec Government to assess community service needs.^21^

For ease of interpretation and consistent with another paper, both dimensions were divided into population quintiles, from quintile 1 (least disadvantaged) to 5 (most disadvantaged), and then dichotomized into “deprived” (quintiles 4 and 5) versus “not deprived” (quintiles 1–3).^22^

#### Perceived Neighborhood Social Cohesion and Disorder

Neighborhood social cohesion and disorder were measured by two scales that have been adapted from the work of Barnes-McGuire^23^ and the Canadian National Longitudinal Survey of Children and Youth.^24^ Both are based on the mother’s perception of her neighborhood. Items forming the social cohesion scale assess the level of agreement to five statements about the support of neighbors, while items on the social disorder scale assess the presence and severity of four types of problems in the neighborhood.^25^ The items for each of the scales can be found in Appendix 1. Scale scores were calculated by averaging item responses for each scale. Social cohesion scores range from 1 to 4, where higher scores indicate a less cohesive neighborhood. Social disorder scores range from 1 to 3 with a lower score indicating the presence of social problems. Both scales were dichotomized to increase interpretability. Social cohesion was dichotomized based on the 50th percentile. For social disorder, children were categorized as either having a perfect score of 3 (no social problems at all in the neighborhood) versus <3 (social problems present). A similar approach was taken by Curtis et al.^28^ in their analysis of neighborhood influences on a variety of health outcomes in a Canadian sample of children. Both cohesion and disorder were available every other data collection cycle starting at cycle 1 (children were 5 months of age) and therefore were analyzed as time-dependent variables (Table 1). Since values were missing for all children at cycle 8 (7 years), the observation at the previous data collection cycle was carried forward (at 6 years) in order to be able to conduct the analysis.

**Table 1:** Description of considered explanatory variables in the QLSCD

Variable	Description	Change over time
Socio-economic/demographic		
Sex	Male yes/no	Invariant
Socioeconomic status (SES)^a^	Calculated based on gross household income, and mother’s and father’s education level, and job prestige; categorized into tertiles—low, middle, and high	Dependent
Mother is an immigrant	Yes/no	Invariant
Single parent family	Yes/no	Dependent
Early life exposures		
Rapid weight gain in infancy	Highest two quintiles of average monthly weight gain from 0 to 5 months	Invariant
Mother smoked during pregnancy	Yes/no	Invariant
Breastfeeding status	Exclusively breastfed to 3 months of age or older; never breastfed; other	Invariant
Birth weight^b^	Low < 2.5 kg; normal ≥ 2.5 kg but ≤4 kg; high > 4 kg	Invariant
Genetic proxies^c^		
Mother is obese	Mother’s BMI ≥ 30 based on self-reported height and weight	Invariant
Overeating phenotype^d^	“Often” eats too much and/or “sometimes” or “often” eats too fast	Invariant
Place		
Materially deprived	Highest two quintiles of the material deprivation factorial score	Invariant
Socially deprived	Highest two quintiles of the social deprivation factorial score	Invariant
High social cohesion	Scale score in the bottom 50 %	Dependent
High social disorder	Less than a perfect scale score (<3)	Dependent
Population density:		Dependent
High	Census metropolitan areas with ≥100,000 inhabitants	
Medium	Census agglomerations with 10,000 to <100,000 inhabitants	
Low	Rural or small towns with <10,000 inhabitants	

Time dependency reflects the fact that these variables were measured at all study time points (4, 6, 7, 8, and 10 years of age) where all available data points were entered into the mixed models analysis. Values for social cohesion and disorder were not collected at 7 years for all children so the value at 6 years was used. For SES and population density, values were not collected at 4 years for all children so the value at 3.5 years (fourth data collection cycle) was used. All time invariant variables were measured at the first data collection cycle of the original cohort study (5 months) except for the genetic proxies

^a^For more information on how this variable was calculated and interpreted, please see Ref. 25

^b^Based on medical records at birth

^c^Recognizing that these factors could also capture elements of the home environment, as well as lifestyle behaviors. Obesity status of the mother might also be considered an early life factor as this was measured when the child was 1.5 years (at the second data collection cycle)

^d^Considered for inclusion based on research showing that at least half of the genetic influence on obesity operates through appetite (see Refs. 26,27; measured at 4 years

#### Population Density

The population density variable was constructed by ISQ by linking participants’ postal codes to census data describing geographical areas using Statistics Canada’s postal code conversion file. According to the linked census information, children were categorized as living in one of four types of geographical areas.^25^ For the purpose of this study, these four categories were collapsed into three, namely, census metropolitan areas containing more than 100,000 inhabitants (high density), census agglomerations containing 10,000 to <100,000 inhabitants (medium density), or rural/small towns containing <10,000 inhabitants (low density). This variable was measured in all data collection cycles (except cycle 5 when children were 4 years of age) and, thus, was analyzed as a time-dependent variable. Because no children had population density collected at 4 years of age, the observation at the previous data collection cycle was carried forward (from 3.5 years).

### Other Explanatory Factors

Other potentially important explanatory factors were identified from recent systematic reviews^29^,^30^ and results of previous studies using the QLSCD.^18^,^31^,^32^ These variables were included in order to gain a more holistic understanding of weight change, as well as to control for potential confounding. A description of these variables is given in Table 1. The mechanism by which explanatory variables could influence weight gain was not the primary focus here. Therefore, factors like lifestyle behaviors (e.g., physical activity, diet, and sleep), family functioning, parenting styles, food security, and general well-being of parents and child were not analyzed in this study as they were considered more proximate mediators.

## Statistical Analysis

In order to achieve the study’s main objective, a growth-curve or random-effects analysis was conducted using PROC MIXED in SAS, version 9.2, using the restricted maximum likelihood estimation method.

First, an exploratory unadjusted analysis was conducted to examine variable distributions and identify outliers and other potential problems with the data. Graphical analysis was conducted to investigate the shape of the BMI Z-score trend and to assess, in an exploratory fashion, the importance of the considered explanatory variables. Time was treated as a continuous variable (age in years) and was centered at the mean (approximately 7 years). To determine the base model from which to conduct further multivariable modeling, four “unadjusted” models, i.e., including only age as either linear, quadratic, cubic, or spline at 7 years, were compared using likelihood ratio tests.^33^ The G matrix was assumed to be factor analytic.^34^ It was determined that the quadratic model fit the data better than the other models and was thus used in further model building. Modeling of explanatory factors involved adding all potential explanatory variables together to the unadjusted model and adding interactions between the explanatory variables and age and age^2^. The interaction terms involving age and age^2^ were reduced by backwards elimination using *α* = 0.05.

The fit of the adjusted model was checked graphically to investigate violations of assumptions about random effects or the specification of fixed effects to identify potential outliers or observations having undue influence on the model and the need to transform particular covariates. Variance inflation factors were calculated for a cross-sectional model of BMI Z-score in order to assess multicollinearity between included explanatory variables. Ethics approval to conduct this analysis was given by the University of Ottawa Research Ethics Board—certificate number H 05-10-18.

## Results

Of the 2,120 cohort children, 1,799 had at least one BMI Z-score measure (out of five possible measures). Because of the cumulative effect of missing observations, 1,580 had complete data on all explanatory variables and could be included in the analysis (75 % of original sample). Almost 43 % of included children had all five BMI Z-score response points, 24 % had four, 12 % had three, 9 % had two, and 12 % had one. The 540 children who were excluded were more likely to be from low socioeconomic status (SES) households, have immigrant mothers, live in materially and socially deprived neighborhoods, and exhibit the overeating phenotype compared to children who were included (*χ* ^2^ *P* < 0.05). Of the excluded children that had response measures, there were no differences in BMI Z-score between excluded and included children at any of the time points (ANOVA *F* test).

Characteristics of included children are presented in Table 2. Both mean BMI and mean BMI-Z score increased over the study period (Table 3). The increasing BMI Z-score demonstrates that, on average, children were getting heavier for their height, age, and sex.

**Table 2:** Baseline descriptive data (4 years) of included children (*n* = 1,580)*

Variable	Percentage (*n*)
Socio-economic/demographic	
Male	50.0 (790)
Socioeconomic status (SES)	
Low	31.9 (499)
Middle	33.9 (530)
High	34.2 (535)
Mother is an immigrant	8.5 (135)
Single parent family	13.2 (208)
Early life exposures	
Rapid weight gain in infancy	38.7 (611)
Mother smoked during pregnancy	24.9 (394)
Breastfeeding status	
≥3 months exclusively	25.8 (408)
Never	27.2 (429)
Other	47.0 (743)
Birth weight	
Low	3.6 (57)
Normal	85.7 (1354)
High	10.7 (169)
Genetic proxies	
Mother is obese	9.9 (157)
Child overeats	22.5 (355)
Place	
Materially deprived	37.9 (599)
Socially deprived	37.1 (586)
High social cohesion	47.8 (732)
High social disorder	26.5 (416)
Population density	
High	66.4 (1035)
Medium	11.2 (174)
Low	22.5 (350)

*Due to missing data on time-dependent variables, denominators for these variables are slightly less than 1,580

**Table 3:** Directly measured mean BMI and BMI Z-score by mean age for included children (*n* = 1,580)

Age (years) (SD)	BMI (SD)	BMI Z-score (SD)	Total *N*
4.2 (0.26)	15.7 (1.60)	0.014 (1.23)	1,352
6.1 (0.25)	15.7 (1.90)	0.031 (1.15)	1,008
7.1 (0.25)	16.1 (2.27)	0.043 (1.10)	1,296
8.1 (0.26)	16.8 (2.59)	0.170 (1.10)	1,161
10.1 (0.26)	18.4 (3.24)	0.342 (0.99)	1,123

*SD* standard deviation

In the unadjusted BMI Z-score trend model (Table 4), the linear and quadratic parameters were positive and statistically significant, indicating that the growth in BMI among these children was accelerating from approximately 4–10 years of age.

**Table 4:** Unadjusted and adjusted BMI Z-score trend models: significant fixed effect parameters and their 95 % confidence limits (*n* = 1,580)

Fixed effects	Parameter estimate	95 % Confidence limits
Unadjusted model		
Intercept	0.062*	0.011, 0.114
Age	0.049***	0.039, 0.060
Age^2^	0.012***	0.008, 0.016
Adjusted model		
Intercept	−0.136 *	−0.268, −0.006
Main effects		
Age	0.031 *	0.006, 0.055
Age^2^	0.013***	0.007, 0.020
Male	−0.116*	−0.213, −0.021
Low SES^a^	−0.123**	−0.217, −0.033
Middle SES^a^	−0.087*	−0.163, −0.014
Rapid weight gain in infancy	0.333***	0.223, 0.444
Mother smoked during pregnancy	0.135*	0.021, 0.249
High birth weight^b^	0.390***	0.236, 0.549
Low birth weight^b^	−0.456**	−0.716,−0.197
Mother is obese	0.686***	0.528, 0.845
Child overeats	0.422***	0.308, 0.537
Materially deprived	0.013	−0.097, 0.124
Socially deprived	0.088	−0.011, 0.188
High social disorder	0.043	−0.025, 0.110
Low population density^c^	0.011	−0.094, 0.116
Medium population density^c^	0.005	−0.123, 0.135
Linear age effects		
Male	0.047***	0.026, 0.068
Low SES^a^	0.050**	0.022, 0.078
Middle SES^a^	0.020	−0.005, 0.046
Rapid weight gain in infancy	−0.009	−0.032, 0.014
Child overeats	−0.026*	−0.052, −0.000
High birth weight^b^	−0.043*	−0.076, −0.009
Low birth weight^b^	−0.001	−0.062, 0.059
Materially deprived	−0.004	−0.028, 0.020
Socially deprived	−0.029*	−0.051, −0.007
High social disorder	0.020	−0.004, 0.044
Low population density^c^	0.002	−0.026, 0.030
Medium population density^c^	−0.050**	−0.083, −0.016
Quadratic age effects		
Rapid weight gain in infancy	−0.009*	−0.018, −0.001
Materially deprived	0.011**	0.003, 0.020
High social disorder	−0.012*	−0.022, −0.002

Model also adjusted for main effects of breastfeeding status, single parent family status, mother’s immigrant status, and high social cohesion (all were not significant)

*P ≤ 0.05; **P ≤ 0.01; *** *P* ≤ 0.0001

^a^Reference is high SES

^b^Reference is normal birth weight

^c^Reference is high population density (census metropolitan area)

**FIGURE 2.**
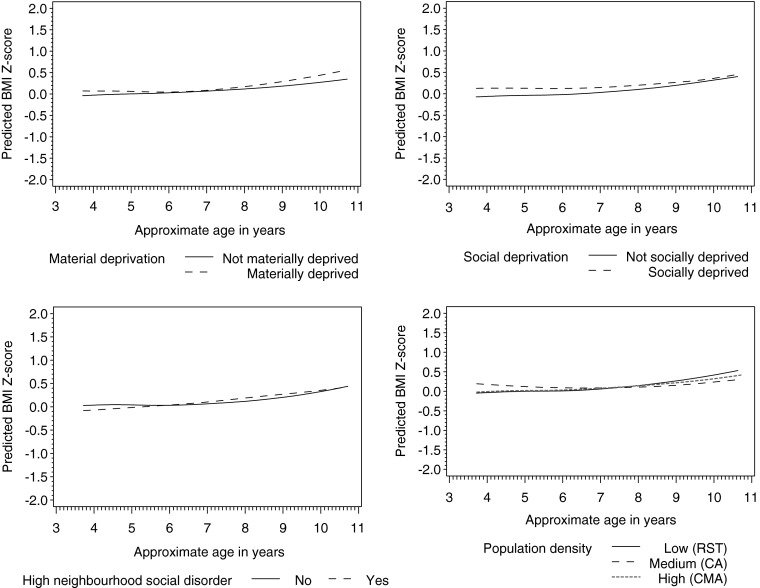
Predicted BMI Z-score smoothed individual trends by significant place factors in the QLCSD, adjusted for other model covariates.

**FIGURE 3.**
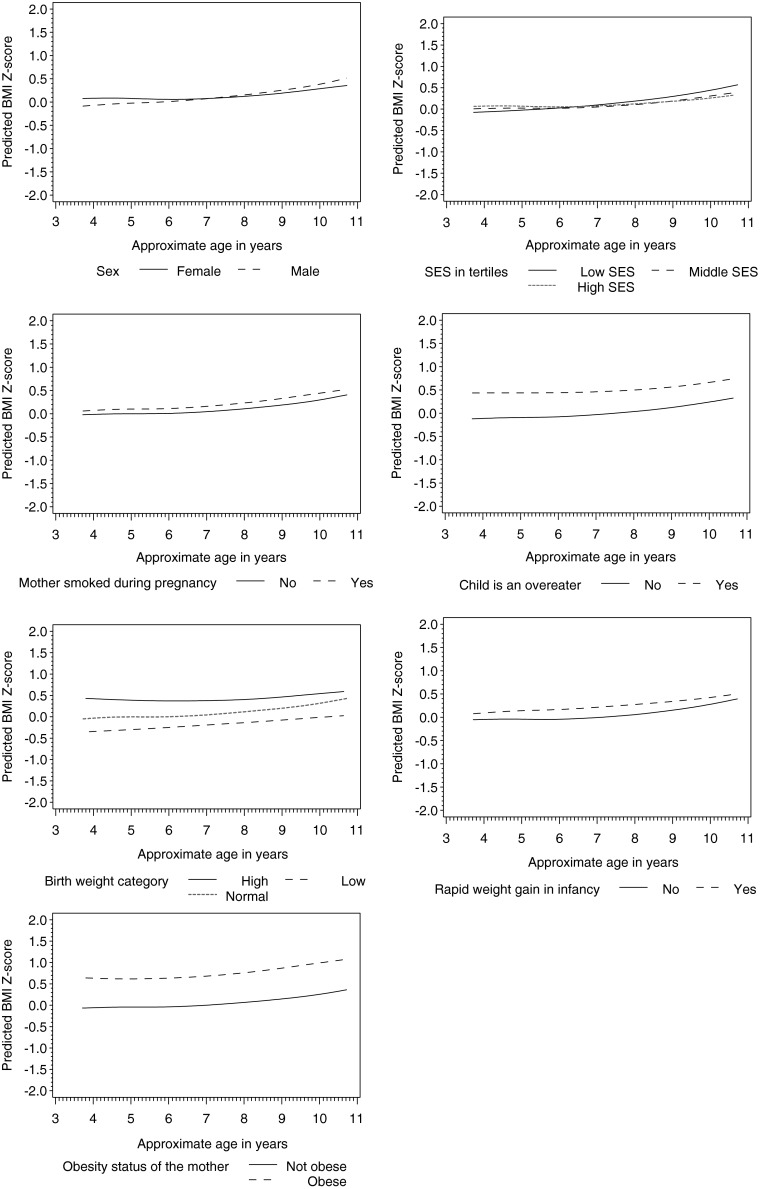
Predicted BMI Z-score smoothed individual trends by significant social and early life factors, adjusted for other model covariates.

The adjusted model is also presented in Table 4. Smoking during pregnancy and obesity status of the mother were significantly related to higher BMI Z-scores on average but did not interact with time. In terms of linear (or uniform) change over time, being male was associated with a faster rate of weight gain compared to being female, as was being from a low SES household relative to a high SES household. Living in a socially deprived neighborhood was significantly related to a slower rate of weight gain compared to nonsocially deprived areas. This association was also seen for living in medium density (census agglomerations) compared to high density areas (census metropolitan areas), between overeaters and nonovereaters, as well as between high birth weight relative to normal birth weight children. In terms of the nonlinear or nonconstant change component of the model (quadratic or age^2^), trends significantly differed for material deprivation, high social disorder, and rapid weight gain during infancy. Specifically, living in a materially deprived neighborhood was related to a higher accelerated weight gain relative to nonmaterially deprived areas. Conversely, living in a high disorder neighborhood was related to a lower accelerated weight gain relative to living in a nondisordered area, as was experiencing rapid weight gain during infancy versus normal growth. A visual example of the associations between significant place factors and weight gain over time is provided in Figure 2. Additionally, Figure 3 shows the differences in trends between levels of significant social and early life variables in the adjusted model.

Using a method developed by Lipsitz et al.,^35^ the final model *R* ^2^ was calculated to be 0.80, indicating good predictive ability. Graphical model checking did not reveal any major violations of statistical assumptions and indicated that the model adequately fit the data. Variance inflation factors did not indicate significant multicollinearity among explanatory factors.

## Discussion

This study sought to examine the influence of place factors on children’s weight status using longitudinal methods, while simultaneously controlling for social and early life factors, as well as genetic proxies. Significant associations were found for material and social deprivation, social disorder, and population density.

This study showed that weight change was positively related to age in this cohort of children, where rates of weight gain accelerated over time. The overall model suggests that early life factors play a role in childhood weight gain. For example, smoking during pregnancy and mother’s obesity status were related to higher BMI Z-scores on average, and even though high birth weight and rapid weight gain in infancy were associated with slightly slower rates of weight change over time, children who had these characteristics had higher BMI Z-scores throughout the study compared to those without (Figure 3). Taken together, these results corroborate those of previous studies in a recent systematic review.^29^

In terms of individual and family characteristics, the difference in trends between males and females found in this study is in contrast to the findings of two other longitudinal studies that did not find significant interactions with time.^11^,^36^ Low SES being related to faster weight gain, on the other hand, has been demonstrated in other longitudinal studies.^37^

For the main explanatory factors of interest, living in materially deprived neighborhoods was related to higher accelerated weight gain. The general positive relationship uncovered here is in line with previous cross-sectional and longitudinal studies.^9^,^38^,^39^ Very few studies examining the effect of neighborhood characteristics on weight in children have actually partitioned neighborhood deprivation into two dimensions, such as was done in this study. Here, the influence of social deprivation was in contrast to that of material deprivation; the rate of weight gain among children who lived in areas with high social deprivation was slower than that of children who lived in nonsocially deprived areas. Using Québec information systems covering mortality, hospitalizations, and births, the creators of the deprivation index used in this study found that the two forms of deprivation had differing impacts on health.^19^ However, a cross-sectional study that used the same index did not find that social deprivation was significantly related to overweight among Canadian adults.^22^

Similar to high social deprivation, living in neighborhoods with high social disorder was related to lower accelerated weight gain relative to not living in such areas. Again, few studies have related this neighborhood characteristic to weight status in children. Rather, studies have examined a similar concept, neighborhood safety, but have met with largely null results.^9^ Even though the findings for high social deprivation and high social disorder are in contrast to the original hypothesis, that they would be positively related to weight gain in children, they are interesting and informative nonetheless, and reflect the complexity of environmental influences on childhood weight status. One explanation for this finding is that such adverse social places may take longer to exert their weight promoting effects. While children are young, on the other hand, they may have the opposite effect—restricting growth, similar to what is often found in the early child development literature.^28^,^40^,^41^ On the other hand, it may be that these environmental influences provide protection from weight gain for reasons not yet understood.

Finally, population density was hypothesized to have a linear relationship with weight status, such that as density increased, weight gain would decrease. The findings here do not corroborate this: Children living in medium density (census agglomerations) areas exhibited slower growth than children in high density areas (census metropolitan areas), and there was no difference between low density (rural/small towns) and high density areas. Overall, the literature in this area is methodologically heterogeneous with similarly mixed results.^9^,^42^ A study conducted by Statistics Canada used nationally representative data to determine the unadjusted regional distribution of child and adolescent overweight. They did not find that the prevalence of overweight significantly differed across census metropolitan areas, census agglomerations, and rural/small towns.^43^

The reason(s) for an inverse association between living in medium density areas and weight status may reflect a more complex reality than the original hypothesis was able to capture. For example, even though census agglomerations are less densely populated than census metropolitan areas, they have an urban core and can act like census metropolitan areas.^44^ Some have high functional metropolitan scores because they act as regional centers and therefore provide a range of services.^44^ Some may not have high functional scores but are located in close proximity to a census metropolitan area, benefiting from services provided close by. Thus, census agglomerations may function as more of a close-knit community than a census metropolitan area, with services close at hand, in contrast to rural/small towns whose residents must commute longer distances to access services and to go to work. The importance of population density on weight gain in children requires further study, and indeed longitudinal findings like these may not corroborate some earlier cross-sectional relationships observed. It is also possible that the relationship between population density and weight may change over time. This could also be said for the other factors considered here.

The findings of the present study should be interpreted in light of some limitations. First, this was a secondary analysis, which limited us to the variables that were collected. For example, material and social deprivation were measured once at the start of the cohort study and therefore could have changed over the study period. The place factors available in the QLSCD provided a 10,000 versus 100 ft view of the place–weight status relationship, as more direct variables such as amenities, infrastructure, etc. were not available.

Due to the study design, some earlier time-dependent covariate data (from 5 months to 3.5 years) could not be accounted for in modeling. The study design also did not permit the use of sample weights. Therefore, the results are not necessarily generalizable to the Québec population. The overall model was fairly simplistic in regards to social–ecological theory. Other settings such as daycare and school were not included, and effect modification was not explored in order to keep the analysis manageable and parsimonious. Consideration should also be given to the importance of the place factors relative to the genetic proxies and factors operating in early life; variables such as obesity status of the mother, overeating, high birth weight, rapid weight gain in infancy, and smoking during pregnancy appear to be more strongly related to weight status than the individual socio-demographic/economic and place factors (see Figures 2 and 3).

Finally, children in this cohort may not follow a homogenous functional form of weight development, which can be characterized by a population-averaged model. This warrants a comparison between the results observed here and those derived from a group-based trajectory modeling approach, such as has been used in previous studies of childhood obesity.^45^–^47^

This study shows that, on average, cohort children were getting heavier over time and suggests the existence of individual and neighborhood social inequalities in childhood weight change. Further work is needed to clarify these relationships, especially for neighborhood-level factors. As in other studies, results here also point to early life as a potentially important developmental window for obesity. Future work, therefore, should also seek to determine the relationship between place characteristics during the perinatal period and these early life risk factors for obesity. Childhood obesity research is, by its very nature, complex. This merits a holistic approach where researchers should continue to focus “upstream” on the interrelationships between different contexts, settings, life stages, and generational transmission, in addition to traditional risk factors such as diet and physical activity.

## Appendix 1

**Table 5:** Neighborhood social scale items

Scale	Response categories
Social cohesion	
Please tell me whether you strongly agree, agree, disagree, or strongly disagree about the following statement…	
1. If there is a problem around here, the neighbors get together to deal with it	Strongly agree = 1
2. There are adults in the neighborhood that children can look up to	Agree = 2
3. People around here are willing to help their neighbors	Disagree = 3
4. You can count on adults in this neighborhood to watch out that children are safe and do not get in trouble	Strongly disagree = 4
5. When I am away from home, I know that my neighbors will keep their eyes open for possible trouble	
Social disorder	
How much of a problem is the following in this neighbourhood…	
1. Litter, broken glass or garbage?	A big problem = 1
2. Selling or using drugs?	Somewhat of a problem = 2
3. Alcoholics and excessive drinking in public?	No problem = 3
4. Groups of young people who cause trouble?	
